# Social cognition in adolescents at risk for psychosis: A 2-year follow-up study

**DOI:** 10.1192/j.eurpsy.2021.604

**Published:** 2021-08-13

**Authors:** L. Pelizza, S. Azzali, S. Garlasdi, I. Scazza, F. Paterlini, M. Poletti, S. Pupo, A. Raballo

**Affiliations:** 1 Department Of Mental Health, Azienda USL di Parma, Parma, Italy; 2 Department Of Mental Health, Azienda USL-IRCCS di Reggio Emilia, Reggio Emilia, Italy; 3 Anrsthesia And Resuscitation Service, Azienda Ospedaliero-Universitaria di Parma, Parma, Italy; 4 Center For Translational, Phenomenological And Developmental Psychopathology (ctpdp), Perugia University Hospital, Perugia, Italy

**Keywords:** Ultra-High Risk, adolescence, social cognition, early psychosis

## Abstract

**Introduction:**

Deficits in social cognition have been reported in people at ultra-high risk (UHR) of psychosis exclusively using socio-cognitive tasks and in adolescent and young adult mixed population.

**Objectives:**

Aim of this study was (1) to assess subjective experience of social cognition in adolescent help-seekers identified through UHR criteria, (2) to explore its significant correlations with psychopathology and functioning in UHR individuals; and (3) to monitor longitudinally its stability after a 24-month follow-up period.

**Methods:**

Participants [51 UHR, 91 first-episode psychosis (FEP), and 48 non-UHR/FEP patients], aged 13–18 years, completed the comprehensive assessment of at-risk mental states and the GEOPTE scale of social cognition for psychosis.

**Results:**

In comparison with non-UHR/FEP patients, both UHR and FEP adolescents showed significantly higher GEOPTE total scores. After 12 months of follow-up, UHR individuals had a significant decrease in severity on GEOPTE “Social Cognition” subscore. In the UHR group at baseline, GEOPTE scores had significant positive correlations with general psychopathology, positive and negative dimensions. Across the 2-year follow-up period, social cognition subscores specifically showed more stable associations with general psychopathology and negative symptoms.

**Conclusions:**

Social cognition deficits are prominent in UHR adolescents and similar in severity to those of FEP patients at baseline. However, these impairments decreased over time, presumably together with delivery of targeted, specialized models for early intervention in psychosis.

